# Unmanned Aerial Vehicle-Borne Sensor System for Atmosphere-Particulate-Matter Measurements: Design and Experiments

**DOI:** 10.3390/s20010057

**Published:** 2019-12-20

**Authors:** Tonghua Wang, Wenting Han, Mengfei Zhang, Xiaomin Yao, Liyuan Zhang, Xingshuo Peng, Chaoqun Li, Xvjia Dan

**Affiliations:** 1 College of Mechanical and Electronic Engineering, Northwest A&F University, Yangling 712100, China; wangtonghua@nwafu.edu.cn (T.W.); 2018050987@nwafu.edu.cn (M.Z.); yaoxiaomin0604@163.com (X.Y.); liyuanzhang@nwafu.edu.cn (L.Z.); pxs@nwafu.edu.cn (X.P.); chaoqun92@nwafu.edu.cn (C.L.); 2 Institute of Soil and Water Conservation, Northwest A&F University, Yangling 712100, China; 3 Department of Civil and Environmental Engineering, Colorado State University, Fort Collins, CO 80523, USA; 4 Nanjing Hepu Aviation Technology Co., Ltd., Nanjing 211300, China; danxujia2008@163.com

**Keywords:** PM2.5, PM10, autonomy and selectivity, three-dimensional stereoscopic monitoring, propeller disturbance, environmental monitoring

## Abstract

An unmanned aerial vehicle (UAV) particulate-matter (PM) monitoring system was developed that can perform three-dimensional stereoscopic observation of PM2.5 and PM10 in the atmosphere. The UAV monitoring system was mainly integrated by modules of data acquisition and processing, wireless data transmission, and global positioning system (GPS). Particularly, in this study, a ground measurement-control subsystem was added that can display and store collected data in real time and set up measurement scenarios, data-storage modes, and system sampling frequency as needed. The UAV PM monitoring system was calibrated via comparison with a national air-quality monitoring station; the data of both systems were highly correlated. Since rotation of the UAV propeller affects measured PM concentration, this study specifically tested this effect by setting up another identical monitoring system fixed at a tower as reference. The UAV systems worked simultaneously to collect data for comparison. A correction method for the propeller disturbance was proposed. Averaged relative errors for the PM2.5 and PM10 concentrations measured by the two systems were 6.2% and 6.6%, respectively, implying that the UAV system could be used for monitoring PM in an atmosphere environment.

## 1. Introduction

With the rapid advancement of urbanization and industrialization, large-scale activities of industrial production and transportation discharge a large amount of air pollutants into the atmosphere, causing widespread public and government attention [1,2,3]. Fine particulate matter (PM2.5) and inhalable particles (PM10) are the primary pollutants. PMs not only cause serious harm to people’s health, but also have an increasingly serious impact on crop production and agricultural-product quality [4,5,6,7]. A larger concentration of fine PM in the air affects plant respiration. Under smoggy weather, poor light conditions can affect plant photosynthesis and crop yield. Meanwhile, smoggy weather can also lead to delays in the growth period of crops, frequent pests and diseases, and the decline in the yield and quality of agricultural products, as well as cause harmful substances to remain in the crops. Eating harmful crop products can indirectly hurt people’s health [8,9,10]. Therefore, large-scale three-dimensional PM measurements are needed to provide decision support for the precise control of atmosphere pollution, and to provide technical support for follow-up studies to resolve PM influence farmland crops.

Presently, traditional PM monitoring methods can be divided into four categories. The first is fixed-point observation, usually with the vertical distribution of PM2.5 mass concentration in the near-surface layer measured from meteorological towers [11,12,13]. The tower, as a relatively traditional observation method, has a wide range of applications. However, it is fixed on spot and difficult to move, with limitations on observation range and height. The second is tethered aerostat observation, with different sensors loaded on tethered balloons to measure vertical distribution of high-level contaminants [14,15,16,17,18]. However, a tethered aerostat cannot flexibly observe in a wide range. The third is the high-altitude observation of a manned aircraft equipped with sensors to measure pollutant concentration. Although a manned aircraft can perform observation at a wide range, its cost is relatively high and it is rarely used for experiment research. The fourth is the remote sensing of low Earth orbit satellites [19,20,21,22,23,24]. Although their detection range is large and the macroscopic distribution of pollutants can be reflected on different time and space scales, observation at a particular time and location is usually limited by satellite orbits, making it difficult to conduct real-time measurement as required to obtain microfine monitoring data.

With the development of modern control and intelligent technologies, the application of unmanned aerial vehicles (UAVs) has received wide global attention. As an observation platform, a UAV is easy to operate, with good stability and high flexibility and security [25,26]. In an environmental emergency, UAVs can quickly overcome the inconvenience of traffic to reach many airspace and/or dangerous areas that cannot be reached by humans where a pollution accident is located, and quickly check the situation. It has good timeliness and provides decision support for the precise management of environmental issues [27]. When a UAV is used for the observation of PM concentration above crops, it provides a better monitoring platform, and can flexibly and efficiently perform large-scale three-dimensional measurement to provide technical support for the study of the subsequent impact of PM on crops.

In recent years, UAV monitoring systems have rapidly developed. On 8–9 December 2015, the meteorological center of China meteorological administration used unmanned helicopters to detect the vertical variation of aerosol mass concentration in Fangshan, Beijing [28]. Peng Z R used the mobile sensor carried by the UAV to monitor PM2.5 concentration in Hangzhou [29]. UAVs can be used for aerial-pollutant measurements, but there are several limitations to existing UAV PM monitoring systems: first, the purpose of most previous studies [30,31] is mainly to integrate sensors into the UAV to acquire, store, and display data in real time. UAV control mode is relatively simple, and measurement frequency and storage mode are fixed and cannot be remotely controlled by the user at real time. For example, Miguel Alvarado et al. integrated photoelectric dust sensor SHARPGP2Y10 and loaded it onto small fixed-wing and multirotor helicopters to collect data during flight, and the measured data could be displayed and stored in real time at the ground station [32], but the system could not perform remote control over the real-time selection of measurement frequency and storage mode. When the UAV system performs large-scale monitoring, the matching between measurement frequency and observation-area and flight-parameter size (flight speed and duration) is very important. When a UAV system is observed under the planned route, the fixed measurement frequency of its predecessors may not be suitable for planning each collection task, and it cannot be accurately monitored at a certain point; especially according to different situations or emergencies encountered at a monitoring site, measurement frequency needs to be replaced in real time, but the system cannot meet the functional requirements. Therefore, the UAV monitoring system still has challenges in terms of function setting. Second, in existing research [33,34,35,36], some studies use 3D scanning and Computational Fluid Dynamics (CFD) technology to simulate and analyze the motion and distribution of the airflow field generated by the drone rotor in an ideal environment, other studies have been tested in the field, so as to find the position of the sensor with less disturbance. The position of the sensor is relatively fixed. However, in existing research, there are few experimental studies on accurate PM values by propeller disturbance in a complex environment. Therefore, in an external environment, the influence of propeller disturbance on measured PM concentration in a UAV monitoring system needs further research.

In view of the above problems, this paper developed a set of UAV monitoring systems that achieve three-dimensional spatial PM observation. A ground measurement-control subsystem was added with better functions for remotely controlling measured-frequency and data-storage modes. The information of PM concentration, geographical location, and time can be viewed in real time through the ground measurement-control subsystem. This study also conducted an experiment to investigate the influence of propeller disturbance on measured PM concentration, while also proposing a method to correct propeller disturbance effect and further improve the practicability of the system. Therefore, the developed UAV system could better provide technical support for the study of atmosphere environments and the impact of PM on farmland crops.

The rest of the paper is outlined as follows: (1) design of the atmosphere PM UAV monitoring system (Section 2); (2) design of observation-mode setting function of atmosphere PM monitoring system (Section 3); (3) integration of UAV PM monitoring system and communication test (Section 4); (4) ground-calibration experiment of onborne PM observation subsystem (Section 5); (5) propeller-disturbance test of atmosphere PM UAV monitoring system (Section 6); (6) application examples of atmosphere PM UAV monitoring system (Section 7).

## 2. Design of Atmosphere PM UAV Monitoring System

### 2.1. Overview

**Figure 1:**
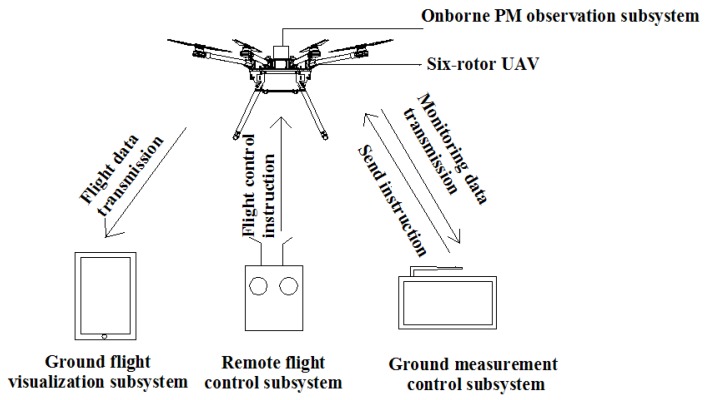
Schematic diagram of unmanned aerial vehicle (UAV) monitoring system for particulate-matter (PM) measurement components: onborne PM observation, six-rotor UAV, ground flight-visualization, remote flight-control, and ground measurement-control subsystems.

The UAV PM monitoring system was mainly composed of a 6-rotor UAV and four subsystems of remote flight control, ground flight visualization, onborne PM observation, and ground measurement control, as shown in Figure 1. The onborne PM observation subsystem primarily measures PM2.5 and PM10, and it was integrated onto the UAV. The flight route and attitude of the UAV are controlled by a remote-flight-control subsystem, and the flight status and performance parameters of the UAV are displayed on the ground flight-visualization subsystem. The onborne PM observation subsystem not only stores measured data in real time, but also enables two-way communication with the ground measurement-control subsystems. The ground measurement-control subsystem can simultaneously display, store, and view the measured PM concentration and the corresponding information of time, latitude, longitude, and altitude for observations. Particularly, this subsystem can remotely and in real time control measurement frequency and storage mode.

### 2.2. UAV Platform

The 6-rotor UAV platform mainly consists of three subsystems: a 6-rotor UAV, remote flight control, and ground flight visualization. From them, the 6-rotor UAV has an FPV aerial photography S550 Printed Circuit Board (PCB) fixed-type 260MM high 3K carbon-fiber-tube-stand 6-axis UAV rack; The DJI N3 multirotor flight-control system was adopted with the latest DJI navigation control algorithm, and the new redundant design of dual IMU can realize real-time data backup. Combined with the new structure design of internal shock absorption, the aircraft is highly reliable. The propeller uses the noise-reduced paddle of PHANTOM 4pro, with a propeller blade of 24 × 13.97 cm in diameter × distance between propellers, and a weight of 11.7 g for each pair of blades. UAV battery is a 10,000 mAh 4S battery. The remote flight-control subsystem uses DJI Datalink 3, and the ground flight-visualization subsystem uses the Apple iPad.

### 2.3. PM Monitoring Platform

**Figure 2:**
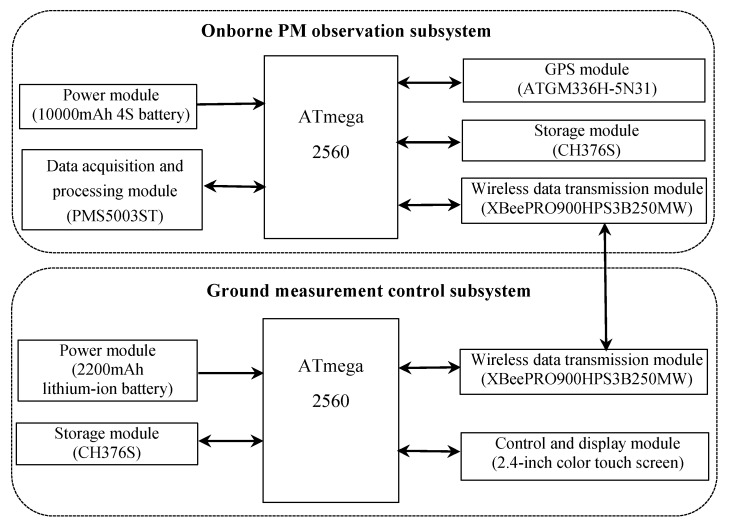
Architecture of PM monitoring module (PM monitoring platform components include onborne PM observation subsystem and ground measurement-control subsystem).

The design of the PM monitoring platform includes chip selection, functional module design, overall schematics, a printed circuit board (PCB) layout, and system-software design. The system is highly integrated to measure PM2.5, PM10, latitude, longitude, altitude, and number of satellites, and enables real-time control of the monitoring system’s monitoring frequency and storage. The PM monitoring platform is mainly composed of two parts: the onborne PM observation subsystem and the ground measurement control subsystem. As shown in Figure 2, the former mainly includes a data-acquisition and -processing module, and a global positioning system (GPS) module; the latter mainly includes a control and display module, each with its own power and storage module, and a wireless data-transmission module. Their functions are discussed as follows.

The onborne PM observation subsystem uses ATmega2560 as the main processor, which is easy to operate, rich in interface, and fast in processing speed; it provides protection for a large amount of data processing. The onborne PM sensor is PMS5003ST, a general sensor for digitalized PM measurement based on the laser-scattering principle. The sensor can be embedded in a variety of instrumentation or equipment for monitoring or improving an environment related to the suspended particulates in the air to provide concentration data accurately in a timely manner. This PM sensor has a small size and it is easy to integrate into a UAV. The GPS module uses an ATGM336H-5N31 component to collect observation-time, latitude, longitude, and altitude information. After the onborne PM observation subsystem completes data acquisition, the subsystem needs to synchronize the GPS with the PM sensor. After collecting all information, all data are converted into a complete data packet, and a specific communication is formed in the whole process. The protocol stores the GPS time, PM2.5, PM10, GPS latitude, longitude, and altitude information for data storage and remote real-time transmission. From them, the storage module uses CH376S for data memory, and the wireless data-transmission module uses the wireless XBeePRO900HPS3B250MW component to transmit data and control commands in real time between the onborne PM observation subsystem and the ground measurement control subsystem. The power module uses a 10,000 mAh battery, and each module provides suitable and stable voltage, which is an important guarantee for the realization of the circuit. After the hardware-circuit design of each module, the software program was designed for the subsystem so that parameters of the onborne PM observation subsystem could be collected and stored in the real-time transmission function.

The ground measurement-control subsystem mainly remotely transmits data from the PM observation subsystem in real time while realizing the real-time setting function of the measurement-frequency and data-storage mode. The main control chip (ATmega2560) controls each module, the power module uses a 2200 mAh lithium-ion battery, the storage module uses CH376S, the wireless data-transmission module uses a wireless XBeePRO900HPS3B250MW component, the control and display module has a 2.4 inch color touch screen that is mainly used to display measurement data and the function and remote-control status of the onborne PM observation subsystem. The monitoring-frequency and data-storage mode can be set by the user according to the user’s requirements. The ground remote-measurement-control subsystem integrates the design of each module and completes the software development of the subsystem, thereby realizing the functions of real-time display, viewing, storage, monitoring-frequency setting, and storage-mode selection.

## 3. Design of Observation-Mode-Setting Function of Atmosphere PM Monitoring System

**Figure 3:**
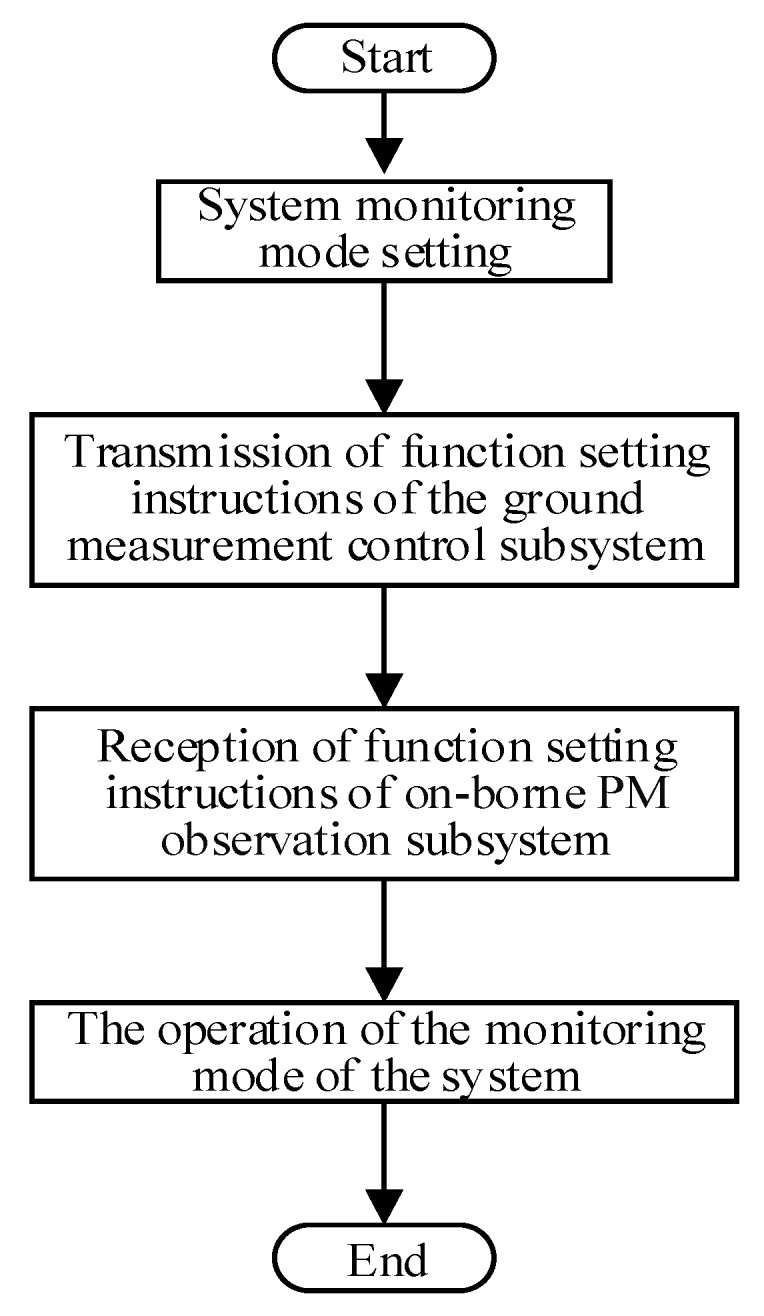
Main system-function-setting flowchart.

Previously existing UAV monitoring systems mainly display and transmit observed data in real time to their ground component [30,31,32,37]. However, the measurement frequency and storage mode of the monitoring system are fixed. Functional requirements of the UAV monitoring system become increasingly higher to further improve the efficiency and autonomy of the UAV monitoring system. When the UAV system is monitored, there is a matching relationship between UAV flight parameters (flight speed and endurance time), and measurement frequency and storage mode. Therefore, the functional modules of the system need to be further optimized. The design of the system measurement-frequency and storage-mode setting function is necessary. In this study, the design of the system setup function was added, which mainly added a new ground measurement-control subsystem that can display, view, and store measurement-frequency settings and storage methods in real time. The ground measurement-control subsystem can perform two-way communication, and not only receive onborne PM observation-subsystem remote-transmission back-data packets, and carry on analysis to it, thus displaying measured data in real time, but also send control instructions, thus remotely controlling the onborne PM observation-subsystem measurement frequency and data storage in real time. System observation is mainly achieved by selecting the control command through the ground measurement-control subsystem, and then performing real-time communication through the wireless data-transmission module on the ground measurement-control subsystem and the wireless data-transmission module on the onborne PM observation subsystem. When the onborne PM observation system receives the signal, control instructions of the onborne PM observation subsystem are invoked to remotely set system functions in real time. The function setting of this system is mainly based on the C language for software design, and the main system-function-setting flowchart is shown in Figure 3.

**Figure 4:**
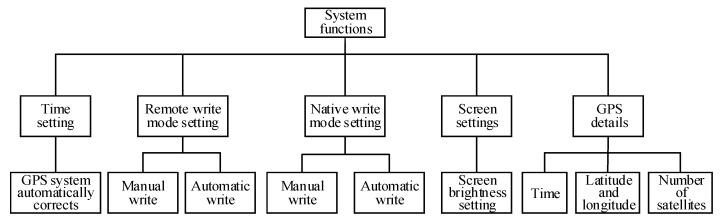
System-function block diagram.

**Figure 5:**
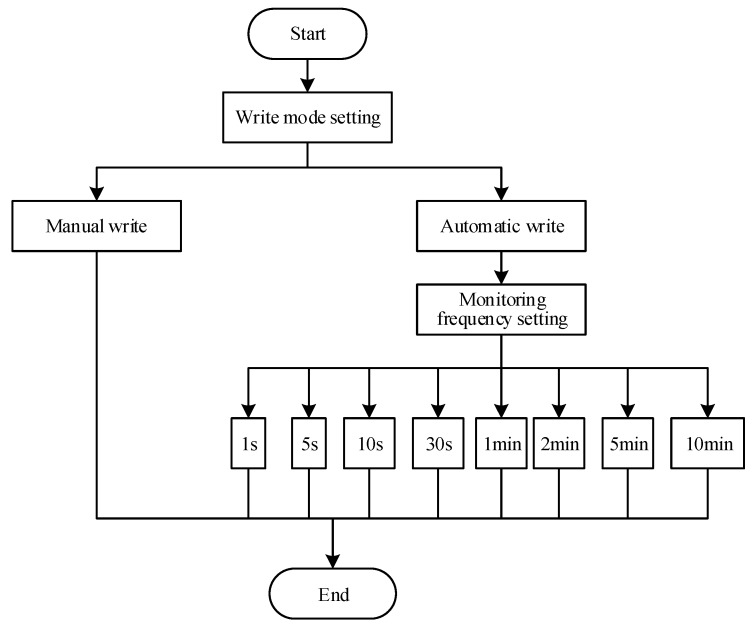
Flowchart of monitoring frequency setting of atmosphere PM UAV monitoring system.

With the increasing functional requirements of the UAV monitoring system, an atmosphere UAV PM monitoring system was developed in order to further improve system function. On the basis of hardware design, the system designs the program of the monitoring system for UAV PM. The user can not only set the measurement frequency, data-storage mode, and screen brightness of the PM monitoring system through the ground measurement-control subsystem, but also view GPS time, latitude, longitude, altitude, and satellite numbers in real time. Therefore, users can receive and view data information of the onborne PM observation subsystem, and control the monitoring function of the UAV PM monitoring system remotely and in real time. The functional block diagram of the system is shown in Figure 4. The storage method of the monitoring data has both manual and automatic writing modes available. The former manually controls data writing; the latter automatically writes data at a preset time interval. Time intervals for data collection can be set by users at 1, 5, 10, or 30 s, and 1, 2, 5, or 10 min as needed. The monitoring-frequency setting of the atmosphere PM UAV monitoring system is shown in Figure 5.

## 4. Integration of UAV PM Monitoring System and Communication Test

**Figure 6:**
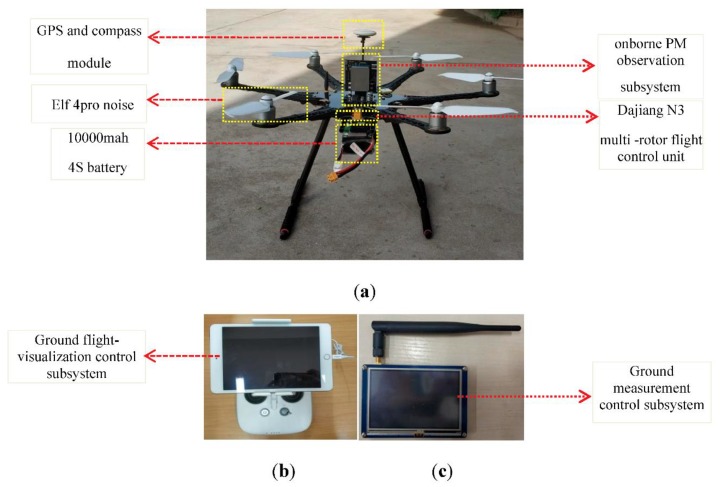
(**a**) UAV PM monitoring system components including onborne PM observation subsystem, Dajiang N3 multirotor flight-control unit, global positioning system (GPS), and compass module, Elf 4pro noise, 10,000 mah 4S battery, and six-axis UAV rack. (**b**) Ground flight-visualization control subsystem. (**c**) Ground measurement-control subsystem.

The subsystems of onborne PM observation, ground flight-visualization control, and ground measurement control were integrated into the UAV. The 10,000 mAh 4S battery was used to support a continuous flight of 20 min after carrying the sensor. On the basis of previous studies’ airflow-field motion distribution, a large number of computational fluid dynamics (CFD) simulation experiments were carried out to determine the position of the sensor [32,33,35]. Propeller disturbance was small at the position directly above the UAV. Therefore, on the basis of previous studies, the onborne PM sensor was located at the center of the six-rotor UAV system, as shown in Figure 6.

On 4 May 2019, vertical-measurement and field-communication tests were conducted at the Institute of water-saving agriculture in arid areas of China (Yangling, Shaanxi). The UAV PM monitoring system was vertically raised (0–500 m) with its measurement frequency set at 1 s. Table 1 shows the measured PM2.5 and PM10 concentrations, and communication success rates at different heights. In particular, in order to prevent data loss and to reduce the measurement error, the system was designed with a checksum and retransmission mechanism to ensure the accuracy and reliability of data communication. It can be concluded from Table 1 that the UAV PM monitoring system had stable performance and good practicability in the 0–500 m height region.

## 5. Ground-Calibration Experiment of Onborne PM Observation Subsystem

**Figure 7:**
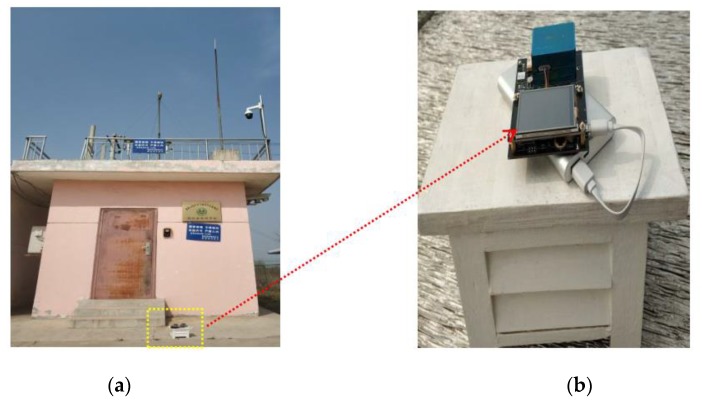
(**a**) Onborne subsystem calibration test at national air-quality monitoring station; (**b**) Onborne PM observation subsystem.

For the developed UAV monitoring system, measurement reliability depends on the onborne PM sensor. First, the sensor was calibrated in the laboratory before leaving the factory. However, after the sensor was integrated into the system, the PM sensor would be affected by actual conditions. Therefore, it was necessary to re-field the integrated system while considering field performance. In previous studies, the monitoring system was examined in field calibration experiments. For example, Man Sing Wong calibrated the monitoring system at the roadside air-quality monitoring station in Mong Kok, Hong Kong [38]. Therefore, this study conducted a calibration experiment at the national air-quality monitoring station (Figure 7).

**Figure 8:**
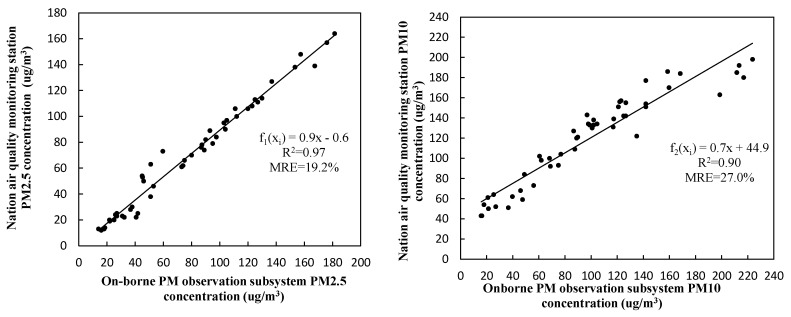
Comparison of PM2.5 (left) and PM10 (right) concentration measurements between onborne PM observation subsystem and standard instrument at national air-quality monitoring station.

The experiment compared *X_i_* and *Y_i_* (*i* = 1, …, *n* is the number of samples) data measured by the onborne PM observation subsystem and by the standard instrument at the national air-quality monitoring station. N = 50 data were sampled to obtain different particle concentrations of PM2.5 and PM10, and their scatter plots are shown in Figure 8. The sampling time intervals were 1 h and 5 min for the data of the national air-quality monitoring station and the onborne PM observation subsystem, respectively; thus, the latter was averaged over a box window of 1 h for comparison. Finally, linear fitting equation *Y_i_* = *f*(*x_i_*), and its coefficient of determination (*R*^2^) and the mean relative error (*MRE*) were obtained, as given by

(1) $$\bar{y}=\frac{1}{n}\sum_{i=1}^{n}y_{i}$$

(2) $$SS_{res}=\sum_{i=1}^{n}{\left(y_{i}-f\left(x_{i}\right)\right)}^{2}$$

(3) $$SS_{tot}=\sum_{i=1}^{n}{\left(y_{i}-\bar{y}\right)}^{2}$$

(4) $$R^{2}=1-\frac{SS_{res}}{SS_{tot}}$$

(5) $$MRE=\frac{1}{n}\sum_{i=1}^{n}\left|\frac{y_{i}-f\left(x_{i}\right)}{y_{i}}\right|\times 100\%,$$

where $\bar{y}$ is the mean value of *Y_i_*.

It can be seen from Figure 8 that linear correlation between the two datasets was relatively good, with *R*^2^ = 0.97 and 0.90 for PM2.5 and PM10, respectively. However, their mean relative errors were large, with *MRE* = 19.2% for PM2.5 and *MRE* = 27% for PM10. Due to the limitation of the sensor calibration before leaving the factory, deviation between PM2.5 and PM10 was quite different. The specific reasons are as follows: (1) Since the laser diode used in the sensor had only one wavelength, the PM sensor was affected by particle concentration and composition. Critical particle composition affected the performance of the laser light-scattering sensor. (2) Light scattering depends on the refractive index of the material, and the light absorption of the material may also affect the light received by the intensity phototransistor. Particle size also directly affects the light-scattering and absorption coefficients. (3) Sensor performance depends on particle composition because light scattering is affected by the refractive index. In summary, PM2.5 and PM10 are different in size, so when a particle is monitored by the laser-scattering principle, the measurement errors of PM2.5 and PM10 are quite different. Laser-scattering PM sensors demonstrated the ability to report PM concentrations with relatively high linearity and moderate reproducibility [39]. In addition, the measurement uncertainty can further reduce the average measurement over a longer period of time. There was also systematic error between both datasets, implying that the data collected by the onborne PM observation subsystem needed to be calibrated.

**Figure 9:**
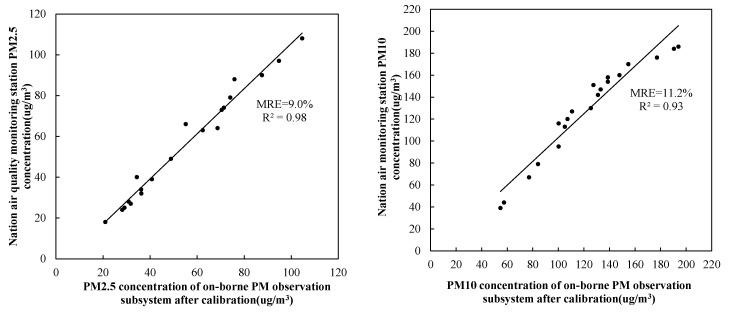
Same as Figure 8, but for the measurements of corrected onborne PM observation subsystem. Comparison of PM2.5 (left) and PM10 (right) concentration measurements between onborne PM observation subsystem after calibration and standard instrument at national air-quality monitoring station.

PM2.5 and PM10 concentrations measured by the onborne PM observation subsystem were corrected and calibrated by using their fitting equations *f*_1_(*x_i_*) and *f*_2_(*x_i_*). For verification purposes, a new dataset of 20 samples was collected by the onborne PM observation subsystem, and calibrated by the fitting equations shown in Figure 8. The calibrated data were compared with the corresponding measurements at the national air-quality monitoring station and are shown in Figure 9.

It can be seen from Figure 9 that the calibrated onborne PM measurements were closer to the national monitoring-station observations, with their *MRE* reduced to 9.0% and 11.2% for PM2.5 and PM10, respectively, within an allowed error range [32], and the reliability of the UAV-measured data could be guaranteed. Differences of around 10% between the two datasets could be attributed to the different principles and accuracy of measurements for the UAV-borne and ground-based sensors. Ground-based sensors have much higher resolution and precision at higher cost [40].

## 6. Propeller-Disturbance Effect on Atmosphere PM Measurement

**Figure 10:**
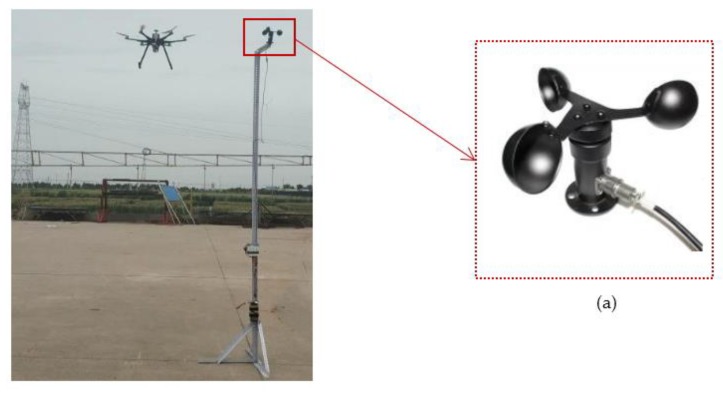
Test for effective distance of propeller-disturbance effect. (a) Three-cup anemometer.

**Figure 11:**
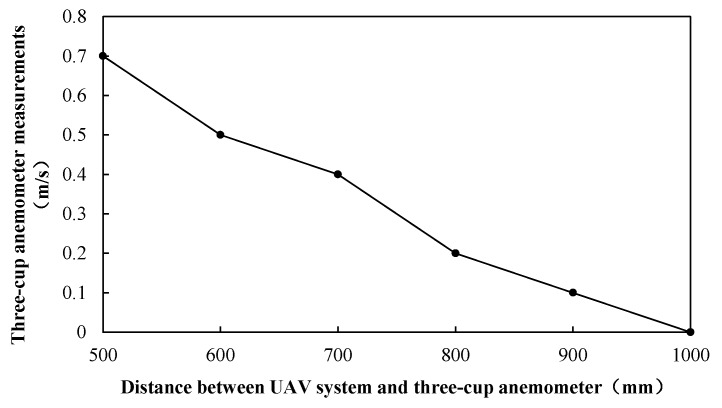
Relationship between wind-speed measurement of three-cup anemometer and distance from hovering UAV system.

When the UAV PM monitoring system is flying, its propeller largely disturbs the air, leading to measurement conditions and results that are quite different from those in the absence of the UAV [34,35,41]. Therefore, an experiment was conducted to test the impact of UAV propeller disturbance on the observation results, and to find a correction method to ensure the reliability of the UAV-corrected data. The existing research [33,34,36] on the influence of UAV propeller disturbance on monitoring concentration is mainly to select a fixed point near the ground to test UAV propeller disturbance, so as to find out the better position of sensor, determine the average relative error of the system, and evaluate the system as a whole, so as to provide support for the system to be better applied to high altitude. In view of the previous studies, the experiment setup included a fixed tower 3.5 m high above the ground at the institute of water-saving agriculture in arid areas of China. A three-cup anemometer was installed at the upper end of the tower to check the disturbance of the UAV propeller (Figure 10). In the absence of outside wind weather, the UAV system gradually moved away from the three-cup anemometer (500, 600, 700, 800, 900, and 1000 mm), and the UAV system hovered at this position for 180 s. After several tests, as is shown in Figure 11, when it was 1000 mm away from the anemometer in the horizontal direction, it was found to have no disturbance effect in the air, consistent with obtained results from previous research [34,36]. In order to determine the minimal distance without a disturbance effect, it provided a basis for the establishment of the following fixed tower.

**Figure 12:**
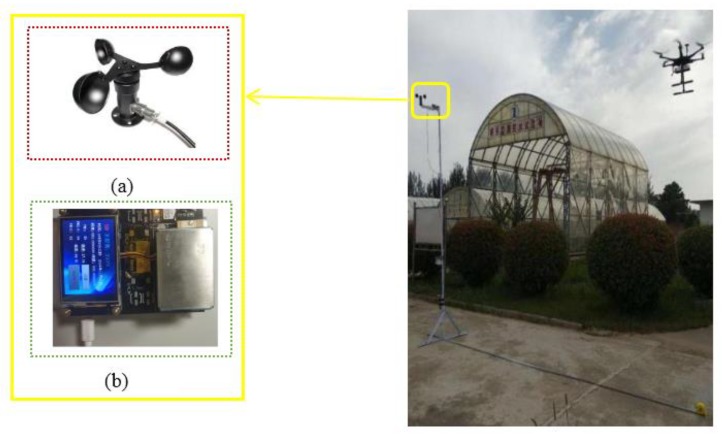
Comparison experiment of fixed tower PM monitoring system and hovering UAV systems. (**a**) Three-cup anemometer. (**b**) Fixed tower data collection unit is an onborne PM observation subsystem.

**Figure 13:**
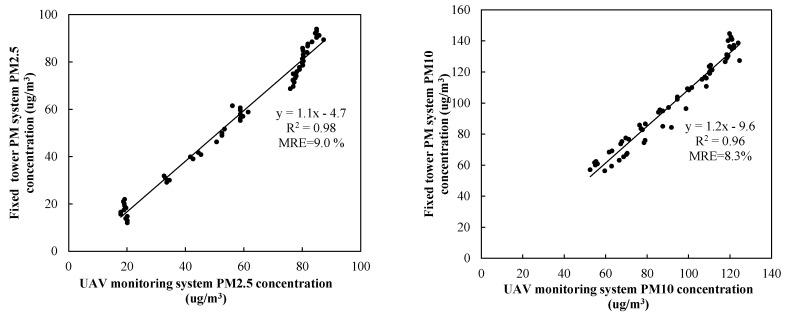
Comparison of PM2.5 (left) and PM10 (right) concentration measurements between PM concentrations measured by fixed and hovered UAV system.

The fixed tower has high stability and is the most traditional type of observation platform. Much previous research was done on the basis of this platform [42,43]. Therefore, the observation on the tower was taken as reference. Note that 1 m was used as the standard for safety distance in order to conduct experiment research on UAV propeller disturbance. Two identical PM monitoring systems were used in our experiment: one was fixed on top of the tower, using its measured concentration as a standard value, and another was integrated into the UAV and hovered at the same height but far greater than 1000 mm away horizontally from the fixed tower for simultaneous measurement (Figure 12). Thus, the relationship between the measurement data of the UAV system and the data on the fixed tower was explored. We obtained two datasets of 60 samples in different environmental conditions (different meteorological factors such as particle concentration, temperature, humidity, wind speed, and pressure atmosphere), each collected by the two systems, and each datum was averaged at intervals of 180 s to obtain different PM concentrations. Measured results are presented in Figure 13 for comparison.

As seen from Figure 13, correlation between the two datasets was high, with *R*^2^ = 0.98 and *MRE* = 9.0% for PM2.5, and *R*^2^ = 0.96 and *MRE* = 8.3% for PM10. Measurements taken by two identical PM monitoring systems at the same time better avoid deviation caused by different ones. However, the two datasets were still biased, indicating deviation mainly caused by propeller disturbance.

A correction method for the propeller effect is proposed as follows. A linear fitting to the measured data was performed to obtain

(6) $$y_{1}=1.1x_{1}-4.7$$

(7) $$y_{2}=1.2x_{2}-9.6,$$

where *x*_1_ and *x*_2_ are concentrations measured by the hovering UAV system for PM2.5 and PM10, respectively, and *y*_1_ and *y*_2_ are corresponding measurements by the fixed tower PM monitoring system.

**Figure 14:**
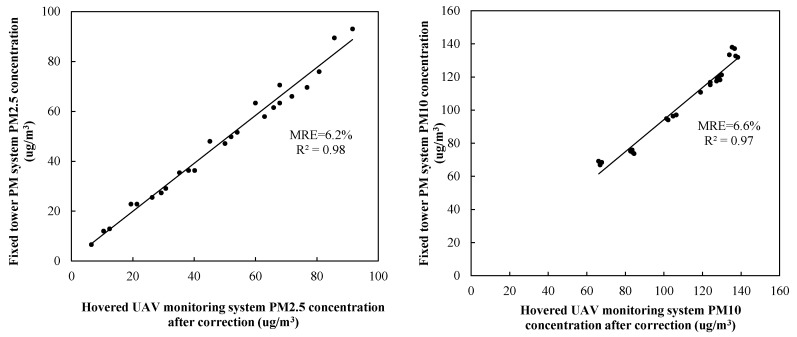
Same as Figure 12, but PM concentrations measured by hovered UAV system were corrected by Equations (6) and (7). Comparison of PM2.5 (left) and PM10 (right) concentration measurements between PM concentrations measured by fixed and hovered UAV system after corrected.

The above-established relations were used to correct the collected data by the hovering system. For this purpose, two new datasets of 25 samples were acquired by the two UAV systems. Equations (6) and (7) were applied to the new data (*x*_1_, *x*_2_) by the hovering system, and to obtain their corresponding results (*y*_1_, *y*_2_), which were then compared with the measured data by the fixed system (Figure 14).

In this experiment, although the propeller disturbance experiment is only carried out at 3.5 m, the experiment is carried out under different weather conditions (different meteorological factors such as particle concentration, temperature, humidity, wind speed, and pressure atmosphere). Based on the experimental study on the influence of propeller disturbance on the observed results, and to find a correction method to ensure the reliability of the UAV-corrected data. It can be seen from Figure 14 that the two datasets had better agreement, with *MRE* reduced to 6.2% and 6.6% for PM2.5 and PM10 concentrations, respectively, implying that to a certain extent, the deviation caused by propeller disturbance of UAV is reduced, so it can be used for monitoring at different altitudes, and so the reliability and practicability of the UAV system were ensured.

## 7. Application Examples of UAV Monitoring System

### 7.1. Vertical-Measurement Cases

**Figure 15:**
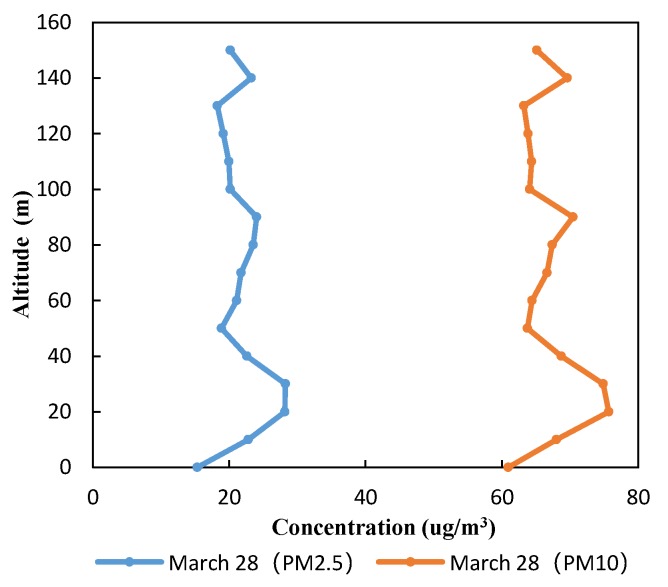
Vertical distribution of PM concentrations on 28 March 2019.

In order to further ensure the practicability of the UAV PM monitoring system, vertical-flight experiments were carried out at the institute of water saving agriculture in arid areas of China. The UAV system measured PM concentrations at different heights from 0 to 150 m above a wheat field, with 3 min hovering and 10 m vertical rise at each height level. Data were collected at a time interval of 5 s and averaged over the 3 min of hovering to obtain PM concentrations at each height. The experiment was started at 16:00 on 28 March 2019, and the measured data were processed offline to obtain the PM concentration profiles shown in Figure 15.

From Figure 15, we can see that PM concentrations presented minima near the ground and some fluctuations with increasing heights, but height variations were relatively small in the range of 0–150 m. The lowest PM concentrations near the surface layer were probably due to the fact that wheat on the experiment field had an adsorption effect on the PM [44,45,46]. PM concentration was the lowest in the vicinity of the surface layer, and the UAV monitoring system could better measure this difference, which further illustrated the practicability of the UAV monitoring system. At heights of 20–30 m, PM2.5 and PM10 concentrations peaked, implying a PM sedimentation layer there, consistent with previous studies [47]. The UAV monitoring system developed in this study could measure the vertical spatial variation of PM concentration and was consistent with previous research results, which further illustrates the practicability and feasibility of the system. For the vertical measurement of PM concentrations near the surface layer, our UAV system provides technical support for air-pollution monitoring with good flexibility, convenient operation, and reliable data, as well as real-time monitoring, in comparison with traditional observations by meteorological towers.

### 7.2. Horizontal-Measurement Cases

**Figure 16:**
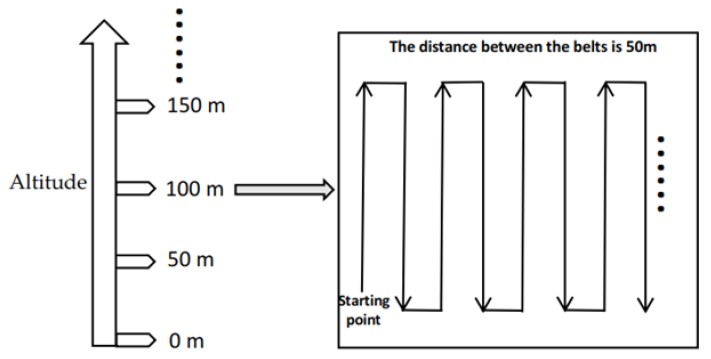
Route plan and flight plan.

**Figure 17:**
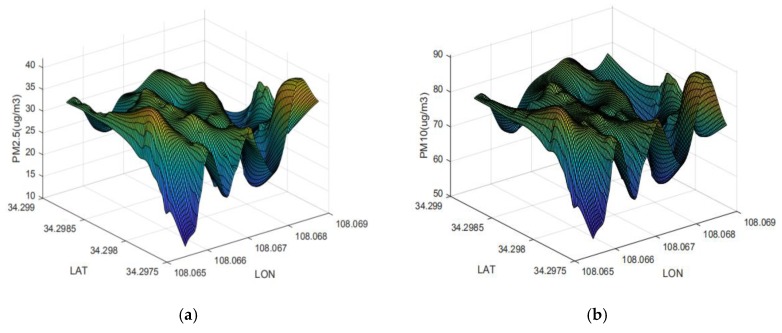
Three-dimensional distribution of PM concentration at institute of water saving agriculture in arid areas of China. Three-dimensional concentration-distribution map of (**a**) PM2.5 and (**b**) PM10.

The UAV PM monitoring system also carried out three-dimensional (height, longitude, and latitude) measurements on the field at the institute of water-saving agriculture in arid areas of China. The system was flown horizontally at a height of 100 m over the wheat field and the persimmon garden. The flight-operation plan is shown in Figure 16. On 9 June 2019, large-scale stereoscopic measurements were taken with a flight speed of 2 m/s; three-dimensional PM distributions were obtained and are shown in Figure 17. Fluctuations of PM2.5 and PM10 concentrations over the observation area were obvious, but variations were small, and the overall shape of the surface was not distorted. Changes were probably due to PM release varying with more vehicles driving by the roads around the test field.

In general, it was feasible for the UAV PM monitoring system to perform stereoscopic measurement on the horizontal plane. The system could flexibly observe PM concentrations at different times, height regions, and horizontal locations. The collected information provides technical support for environmental and crop monitoring.

## 8. Discussion

On the basis of previous research and the increasing demand for UAV monitoring systems, a UAV monitoring system for atmosphere PM was developed that is capable of the flexible and efficient stereoscopic monitoring of PM concentrations. In particular, a ground measurement-control subsystem was designed that can display and store monitored particle concentrations, geographical locations, time, and satellite number, and set the monitoring frequency of the UAV monitoring system, in real time. The data-storage mode can automatically or manually be set by autonomous selection. These features greatly improve previously used UAV systems, whose sensors were mainly integrated onto a drone, and the collected information was transmitted and stored remotely and in real time to a PC. For example Roldán JJ et al. designed a drone-based environmental monitoring system that measures temperature, humidity, luminosity, and CO_2_ concentrations, and exchanges data over a Wi-Fi network [33]. Miguel Alvarado et al. integrated sensors for temperature and humidity (SEN51035P) and dust (GP2Y10) into a drone that transmit the data back to the ground station, enabling real-time display and the recording of the monitored data [34]. In contrast, our system has better autonomy and selectivity, and provides users with more convenient services.

In this study, the selected onborne sensor was PMS5003ST, and we performed a calibration test. After calibration, the *MRE* of PM2.5 and PM10 was 9.0% and 11.2%, respectively, with respect to the standard measurements at the national air-quality monitoring station. The measurement precision of our system was comparable with those of the previous studies. For example, a PM2.5 measurement system developed by Niu Ji used an optical method calibrated with concentration sensor BGPM-02, resulting in an *MRE* of about 5% [48], which was better than ours. However, the GP2Y10 sensor used by Miguel Alvarado et al. was calibrated with a standard air chamber, showing an *MRE* of 14.9% [32], which was slightly worse than ours. The reasons for *MRE* system deviations may be as follows: first, onborne sensors SM-PWM-01A, GP2Y10, and PMS5003ST have different accuracy levels. Second, reference instruments for the calibration are also different, with different accuracy levels. Finally, the experiment environments for the calibration tests were also different, either indoors (Niu Ji) or in a standard air chamber (Miguel Alvarado), in contrast to our field test under different weather conditions of wind speed, temperature, and humidity. In this study, a PM sensor on the basis of the principle of laser light scattering was selected. Previous studies [39,49,50] showed that: (1) similar to organic components, the water absorption of infrared radiation, and due to the reduced light intensity received by the phototransistor, PM concentration is overestimated. (2) High concentrations of humidity may cause circulation circuits to malfunction. The PM sensor causes a bias in the measurement results. (3) When relative humidity is as high as 68%, the PM may deliquesce. PM hygroscopicity (usually affected by the weather) may be a cause of uncertainty in sensor measurement data. (4) Extreme temperature conditions may affect the reported particulate concentration because the flow rate of the particulate ascending airflow and the sensor are determined by the temperature difference between thermistor and environment. (5) Wind speed also affects sensor monitoring. Differences in wind size and direction affect PM spatial distribution, which also affects sensor measuring. In summary, the calibration environment is different and affects the calibration results of the system. All in all, all three factors, alone or together, may cause differences in the calibrated measurements.

When the UAV system is in flight operation, propeller rotation disturbs the air and affects the observed concentration; it was necessary to correct the effect. This study established a comparison test between two identical UAV systems, with one fixed on top of a tower and another hovering in the air under different particle concentrations. The *MRE* between the measurements was PM2.5 (9%) and PM10 (8.3%), respectively. A linear-regression relation between the two datasets was established and used to correct the propeller effect. The corrected data reduced the *MRE* to PM2.5 (6.2%) and PM10 (6.6%). These appeared to be larger than the *MRE* = 3.84%, 3.71% and 1.65% for CO_2_, temperature, and humidity measurements, respectively, reported by Roldán JJ et al. [33]. Differences may be due to the following aspects: (1) differences between six-rotor and quadrotor UAVs; (2) different objects monitored by the systems; (3) differences between our and Roldán JJ’s experiment methods. In the study of Roldán JJ, measurements were taken at a distance of 5 m from the sources at intervals of 1 m. The flying height of the quadrotor drone was 0.5 m. The drone was moved by hand, impacting the observation results. In contrast, our experiment had a fixed tower, and better excluded the influence of manual operation on the measurement results; (4) in the experiments of Roldán JJ, automatic UAV movement was carried out, but not synchronized with the manual one, and the time lapse may have caused deviation. In contrast, our experiment was equipped with the same monitoring system and measured at the same time, better avoiding deviation caused by time asynchrony. In view of these obvious differences, in order to enable the UAV system to carry out flight monitoring more accurately, meteorological and environmental parameters should be taken into account for multiparameter modeling analysis and correction, so as to further improve the monitoring accuracy of UAV monitoring system.

## 9. Conclusions

The UAV system for atmosphere PM monitoring has good application. This study designed a set of the system including a six-rotor UAV, an onborne PM observation subsystem, and ground measurement-control subsystem. The system has a three-dimensional stereoscopic function, and can remotely transmit, store, and display measurement data, and altitude, latitude, longitude, and observation-time information in real-time. In particular, the ground measurement-control subsystem can not only select and set the storage mode of the observed data, but also automatically set the measurement frequency of the system. The system was stable in communication from 0 to 500 m. The system was calibrated by measurements of the national air-quality monitoring station. At the same time, a UAV propeller-disturbance test was carried out, and a method of correcting the propeller-disturbance effect was proposed. The *MRE* between the corrected and referenced data of PM2.5 and PM10 concentrations was reduced to 6.2% and 6.6%. Finally, flight-application tests in vertical and horizontal directions were respectively carried out. The system worked stably, and it can provide technical support for the study of atmosphere environment monitoring and subsequent research on the impact of PM on crops in a farmland environment.

## Figures and Tables

**Table 1:** PM concentration and communication success rate of UAV measurements at different height levels.

Height (m)	PM2.5 Concentration (μg/m^3^)	PM10 Concentration (μg/m^3^)	Communication Success Rate (%)
50	79	128	100
100	81	125	100
150	79	126	100
200	87	126	100
250	97	139	100
300	85	134	100
350	80	129	100
400	82	129	100
450	88	130	100
500	91	139	100
